# A health care labyrinth: *perspectives of caregivers on the journey to accessing timely cancer diagnosis and treatment for children in India*

**DOI:** 10.1186/s12889-019-7911-x

**Published:** 2019-12-02

**Authors:** Neha Faruqui, Rohina Joshi, Alexandra Martiniuk, Jennifer Lowe, Ramandeep Arora, Huma Anis, Manas Kalra, Sameer Bakhshi, Ananya Mishra, Ayyagari Santa, Sudha Sinha, Sirisharani Siddaiahgari, Rachna Seth, Sarah Bernays

**Affiliations:** 1 Sydney School of Public Health, The University of Sydney, Sydney, NSW Australia; 2 George Institute for Global Health, Sydney, NSW Australia; 3 Faculty of Medicine, University of New South Wales, Sydney, Australia; 4 Dalla Lana School of Public Health, University of Toronto, Toronto, Canada; 5 Cankids…Kidscan, New Delhi, India; 6 Max Super Speciality Hospital, New Delhi, India; 7 Indraprastha Apollo Hospital, New Delhi, India; 8 IRCH, AIIMS Hospital, New Delhi, India; 9 Basavatarakam Indo American Cancer Hospital, Hyderabad, India; 10 MNJ Cancer Hospital, Hyderabad, India; 11 Rainbow Children’s Hospital, Hyderabad, India; 12 AIIMS Hospital, New Delhi, India; 13 London School of Hygiene and Tropical Medicine, London, UK

**Keywords:** Qualitative study, India, Childhood cancer, Accessing care, Referral pathways, Treatment delay, Diagnosis delay

## Abstract

**Background:**

Cure rates for children with cancer in India lag behind that of high-income countries. Various disease, treatment and socio-economic related factors contribute to this gap including barriers in timely access of diagnostic and therapeutic care. This study investigated barriers to accessing care from symptom onset to beginning of treatment, from perspectives of caregivers of children with cancer in India.

**Methods:**

Semi-structured in-depth interviews were conducted with caregivers of children (< 18 years) diagnosed with cancer in seven tertiary care hospitals across New Delhi and Hyderabad. Purposive sampling to saturation was used to ensure adequate representation of the child’s gender, age, cancer type, geographical location and socioeconomic status. Interviews were audio recorded after obtaining informed consent. Thematic content analysis was conducted and organised using NVivo 11.

**Results:**

Thirty-nine caregivers were interviewed, where three key themes emerged from the narratives: time intervals to definitive diagnosis and treatment, the importance of social supportive care and the overall accumulative impacts of the journey. There were two phases encapsulating the experiences of the family: referral pathways taken to reach the hospital and after reaching the hospital. Most caregivers, especially those from distant geographical areas had variable and inconsistent referral pathways partly due to poor availability of specialist doctors and diagnostic facilities outside major cities, influence from family or friends, and long travel times. Upon reaching the hospital, families mostly from public hospitals faced challenges navigating the hospital facilities, finding accommodation, and comprehending the diagnosis and treatment pathway. Throughout both phases, financial constraint was a recurring issue amongst low-income families. The caregiver’s knowledge and awareness of the disease and health system, religious and social factors were also common barriers.

**Conclusion:**

This qualitative study highlights and explores some of the barriers to childhood cancer care in India. Our findings show that referral pathways are intrinsically linked to the treatment experience and there should be better recognition of the financial and emotional challenges faced by the family that occur prior to definitive diagnosis and treatment. This information would help inform various stakeholders and contribute to improved interventions addressing these barriers.

## Background

Access to timely diagnosis and treatment for childhood cancers in the Indian health care system is often riddled with multiple barriers [1]. Parents of sick children carry the burden of caring for the child, themselves and the rest of the family while trying to unscramble the fastest and most appropriate pathway to cure. This effort is often incessant when the disease is serious and requires long term treatment. Childhood cancer is such an example, where the disease nature necessitates timely diagnosis and treatment and is the fifth leading cause of death in India amongst 5–14 year olds [2].

‘Access to health care’ has been defined in various ways using common theoretical frameworks [3, 4]. Levesque et al. (2013) for example, conceptualised the term as not only the accessibility of services provided, but the patient’s ability to seek care, pay for it, reach it and engage with the system [5]. Hence, multiple determinants influence the journey to appropriate care via the supply-side of health service provision, organisation and cost of services, and the demand-side of disease burden, patient/caregiver knowledge and attitude [5].

One method used to understand and quantify the challenges to accessing care for childhood cancer is evaluating the time taken to diagnosis (and occasionally time to start treatment) from the time of symptom onset. These time intervals have mostly been derived using framework models by Andersen (1995) [6] and Walter et al. (2012) [7] attributing delays to patient and health system related factors. Our current understanding about delays associated with childhood cancer is obtained mainly from research in high income countries (HICs) [8–11]. Some Indian studies have quantified the time intervals to diagnosis for various childhood cancers such as retinoblastoma, leukaemia and meningioma, where intervals ranged from 0.3 to 521 weeks [12–14]. Reasons for such delays in India included caregiver education level, geographical distances, job demands of the caregiver and multiple healthcare referrals, which were mainly investigated through questionnaires [15, 16]. These provide useful information but lack in-depth analysis about the interplay of barriers in relation to participant's feelings, attitudes and behaviours.

Qualitative studies on access to childhood cancer diagnosis and treatment are rare in low and middle income countries (LMICs), where contextual factors such as cultural beliefs in seeking alternate care vs allopathy (appropriate care), are likely to be more common to cancer care pathways than in HICs. Renner and McGill (2016) conducted a study in Ghana using a social constructionist approach to explore factors influencing parent decision-making for their child with cancer [17]. Themes emerging from this study included lack of awareness and knowledge, seeking traditional medical treatment, need for psychosocial support and factors related to health financing [17]. A similar study was also conducted by Buckle et al. (2013) in Uganda and Kenya, to identify health care seeking behaviours and factors preventing diagnosis and treatment, where barriers included financial costs, transportation issues and household responsibilities [18].

Of the few qualitative studies regarding cancer care in India [19–21], the focus has not been on paediatric oncology and the caregiver’s perspective, although the barriers identified were similar to studies mentioned above. Therefore, this qualitative study aimed to enrich the existing literature by interviewing caregivers of children with cancer and understanding their perspective on barriers to accessing care, which may be at the personal level or health system level. It was not designed to evaluate the strengths of the healthcare system nor the facilitators to accessing care.

## Methods

This study was conducted in north (New Delhi) and south India (Hyderabad) as the lead study investigator (NF) and interviewers were comfortable with the languages spoken in these cities (Hindi, Telugu and Urdu). Although both are large urban metropolitan cities, they are different as Delhi caters to patients from loco-regional as well as distant areas and Hyderabad caters mostly to patients from loco-regional areas. We aimed to recruit hospitals representing the public sector (*n* = 3), private sector (*n* = 3) and charitable trust sector (*n* = 1).

### Sampling and recruitment

Stratified purposive sampling was conducted across 7 hospitals (4 in Delhi and 3 in Hyderabad). Table 1 shows the inclusion and exclusion criteria used. We also aimed to ensure adequate representation of individuals representing but not limited to the following categories which are common in both cities: those with a girl child with cancer (due to potential of gender bias), families travelling from other states (to ensure family experiences were not limited to the local cities), those who are below poverty line (BPL) and children with common cancers (e.g. leukaemia) as well as children with specific tumours (brain, bone and eye tumours). This purposive sampling was conducted to ensure barriers identified were not biased only towards those children and families who would be the easiest to recruit. At the time of the interview, baseline data were collected for caregivers and children for age, sex, cancer type, caregiver relationship and home location. The local principal investigator who was the lead treating oncologist of each hospital, identified patients through their patient list and invited caregivers to participate in the presence of the lead study investigator. The interviewer and lead study investigator then privately described the study purpose to caregivers with the participant information sheet before written informed consent was obtained. It was encouraged that only primary caregivers directly involved in the day-to-day care of the child participate. All interviews were conducted within hospital settings, and depending on room availability some were conducted privately while others were conducted in the wards. To avoid discussion of a sensitive topic around children, arrangements were always made to leave the child either with another caregiver or nurse.

**Table 1:** Inclusion and exclusion criteria applied to the selection of participants

Inclusion criteria:	
• Children diagnosed with cancers before the age of 18 years	
• Diagnosed children who commenced first line treatment (curative or palliative intent) at the participating treating hospital, no more than one month prior to date of recruitment	
• Children residing in India at the time of onset of symptoms and have not travelled outside India to seek care	
• Children who have a caregiver present at the time of the interview	
• Caregivers who can speak the local language or a language known to the interviewers (Hindi, English, Urdu, Telugu)	
Exclusion criteria:	
• Non-malignant haematological conditions like thalassemia, haemophilia	
• Those who have presented with relapse during/after treatment	

### Data collection

All interviews were in-depth and semi-structured conducted by an interviewer with tertiary qualifications in the public health field, who was trained by a local psychologist (HA) and the lead study investigator. The interviewer who was fluent in the local language was accompanied by the lead study investigator for supervision and note-taking in each interview. Both researchers ensured the topic guide was used throughout the data collection process to avoid any interjection of personal bias. Questions were designed based on the trajectory of the journey from symptom onset to diagnosis and treatment. However, being semi-structured, participants were free to narrate incidents in no specific chronological order. Family socioeconomic status (SES) was ascertained through participant narratives. Participants were asked to explain their occupation (if none, occupation of the primary income earner in their household was recorded), an approximate monthly income for those who wished to share and those with a BPL card. Based on this information, SES was divided into those of BPL, low SES and those of middle and/or upper SES (white-collar jobs). Data was collected from various participants until no new conceptual insights were developed in the iterative inductive coding stage, and theoretical saturation was reached.

### Data analysis

All participants were de-identified prior to analysis. Interviews were transcribed and translated into English and NVivo 11 software was used to manage and code the data. For interviews where caregivers wished to be interviewed in pairs, their responses were analysed together. Donabedian (1988) and Penchansky’s (1981) frameworks were initially used to develop a set of broad deductive codes pertaining to barriers influencing access to care [3, 4]. NF and JL independently identified common barriers from literature to further develop a set of barrier themes (codes), which were then classified into sub-themes (sub-codes) (Additional file 1: Table S1). NF and JL compared and discussed the identified sub-codes to categorize them into a final set of deductive codes related to individual, health system and disease barriers. An iterative and inductive approach was then used with content analysis, where new emerging barriers were also coded and gradually a “concept driven” perspective [22] was applied to develop themes. Content analysis was conducted by one researcher who also collected data (NF) and triangulated with iterative suggestions and analytic inputs from a second researcher who was not involved in data collection (SB). In addition, emerging analysis was also independently discussed with RJ and AM as part of the analysis process. Given the emotive nature of the research topic, an important element of the data collection and analysis was to ensure that potential biases were minimised. This involved firstly supporting the researchers involved through close and ongoing supervision to manage the emotional intensity of the research process. Secondly, we adopted an explicitly transparent approach to the analysis and selection of extracts, where narratives were first discussed amongst researchers and described in analytical memos to identify the themes. The researchers then provided their own interpretation of the narratives as well as which extracts were most appropriately suited to the theme. These interpretations were then further discussed between researchers till a consensus was reached. This ensured that the patterns of data were appropriately reflected in the final write-up.

## Results

There were two critical phases in the family’s journey from symptom onset to the start of treatment: referral pathways taken to get to the treating hospital, and care after reaching the hospital. Table 2 shows three inter-related themes and their sub-themes which emerged from the data analysis. Thirty-nine caregivers were interviewed (15 in Hyderabad and 19 in New Delhi, with 6 interviews done in pairs) consisting of 17 fathers, 13 mothers, 7 uncles, 1 sister and 1 cousin. Interview duration in Hyderabad generally ranged from 9 to 20 min and 15 to 63 min in Delhi. Barriers identified in both cities did not largely differ and hence results are presented collectively across participants. Twenty-one caregivers were of lower SES and BPL and 13 from middle-upper SES. Patient sample consisted of 16 females and 18 males, aged between 6 months to 16 years. They were diagnosed with the following cancers: Acute Lymphoblastic Leukaemia (ALL), Acute Myeloid Leukaemia (AML), Non-Hodgkin Lymphoma, Brainstem glioma, Medulloblastoma, Neuroblastoma, Retinoblastoma, Wilms tumour, Rhabdomyosarcoma and Primitive Neuroectodermal Tumour (PNET). Distance to the treating hospital for families residing outside Delhi or Hyderabad was between 15 km – 1800 km, although total distance travelled could be greater due to varied referral pathways. More detail of demographics can be found in Additional file 1: Table S2.

**Table 2:** Themes and sub-themes derived from data analysis

Themes	Sub-themes
1. **Time** (time interval to diagnosis and treatment)	1.1 Efforts and avenues taken in the referral pathway
	1.2 Determinants influencing the referral pathway
	1.3 Patient navigation at the treating hospital
2. **Importance of social supportive care** (facilitated or depleted support in addition to medical care)	2.1 Religious and cultural beliefs
	2.2 Family and social dynamics
	2.3 Ability to stay while receiving treatment
	2.4 Health care provider-patient support
3. **Accumulative impacts of the journey** (overall impacts upon families)	3.1 Financial impacts
	3.2 Ongoing emotional and psychological impacts of the pathway

### Time: stop, slow down or speed up

The three sub-themes defined in this section were all crucial components which impacted upon the time taken to diagnose and treat the child. Sub-theme 1.1 refers to the multiple efforts and avenues undertaken by caregivers in visiting health care providers in the referral pathway before reaching the participating treating hospital. These efforts were influenced by determinants (1.2) such as suggestions by third parties or past experiences within the health system, and paucity of caregiver and health professional knowledge and awareness of childhood cancer. Upon reaching the treating hospital, families then spent time navigating their way through the hospital (1.3), which was also a contributor to time taken to receiving definitive diagnosis and care.

#### Efforts and avenues taken in the referral pathway

**Fig. 1:**
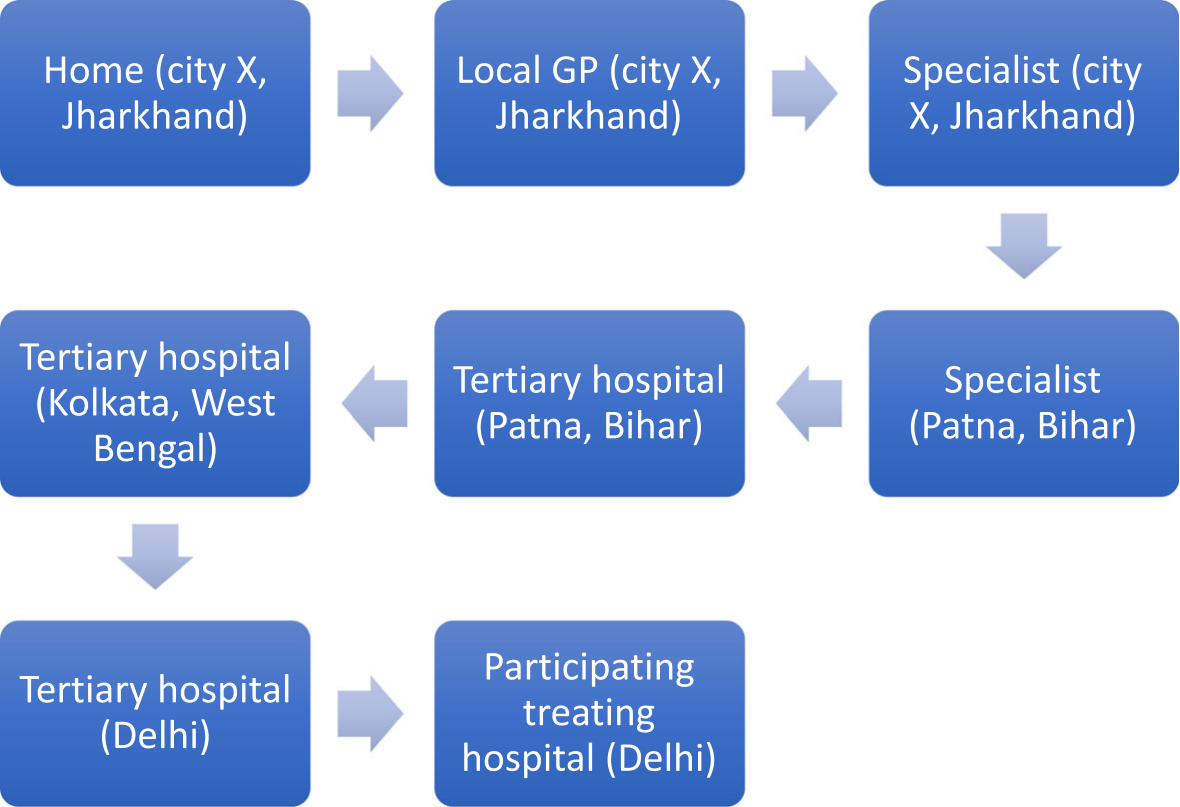
Typical referral pathway for most families traveling from other cities. This journey is an example of a child diagnosed with ALL in Delhi, belonging to a village in the state of Jharkhand who then travelled to cities within the states of West Bengal and Bihar before arriving in Delhi. Note: some of these pathways consisted of repeated visits

**Fig. 2:**
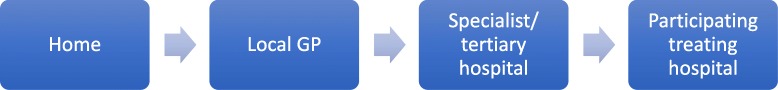
Typical referral pathway for most families residing within the same city as the participating treating centre. This journey is an example of a child diagnosed with AML in Hyderabad and a child diagnosed with Non-Hodgkin Lymphoma in Delhi. Note: some of these pathways consisted of repeated visits

Families took multiple efforts and avenues to navigate the referral pathways. As shown in Fig. 1, those residing outside Delhi/Hyderabad had more complex pathways compared to those living within the city (Fig. 2). However, a few participants, residing within the city also experienced more erratic referral pathways consisting of complex visits. The first time interval occurred during the symptom onset stage. More prominent symptoms such as nose bleeds or blood in the urine prompted the family to seek immediate medical care, while some children with symptoms of fevers, runny nose or cough were treated at home with general medicines, postponing a visit to the doctor. Misdiagnoses such as: dengue, jaundice, rheumatoid arthritis, depression, typhoid, flu, chicken pox, pneumonia, diarrhoea and ulcers, also contributed to delay in reaching the cancer diagnosis. Recurring symptoms then triggered families to seek care with additional health care providers, including traditional healers and rural medical practitioners (RMP).

Even though a few of the regional tertiary hospitals in north and south India were supposed to be able to treat children with cancer, in reality some basic services required were unavailable hence families were referred on. Caregivers often did not question the referrals, however some participants reported that health care providers explained the reason as being a lack of essential diagnostic facilities. Sometimes, caregivers themselves sought other avenues of care due to long admission waiting times or lack of money. One caregiver (Fig. 1) from the state of Jharkhand (approx. 1200 km from Delhi) visited a paediatrician in Patna (state capital of Bihar, approx. 300 km from Jharkhand) three times before being referred to a tertiary hospital in Kolkata (state of West Bengal), where he recounts his experience:

*The senior doctor said that I cannot treat your child unless you deposit Rs. 8 lakhs right now. I said ma’am I cannot deposit so much money now and the NGO said it would be better if you go to Mumbai. At that time I started thinking how do I go to Mumbai? (…) So I told her that ma’am you are sending us away at 5 o’ clock. You could have told us this same thing the day we came here so that we could have left immediately. Now at 5 in the evening where will I go? So I started crying. I said that how much ever cash I had with me was already spent. So how will I go? (Father of child with ALL)*

Another father also described his confused and exhaustive experience of multiple referrals to a paediatrician, orthopaedic surgeon, haematologist and oncologist. Similarly other caregivers acknowledged the delay in timely diagnosis:

*The thing is that ma’am, we should have gone to the bigger hospital right at the beginning so that all the checkup is done. We wasted 3-4 days by going to a smaller clinic. So maybe if we would have gone to a bigger hospital then directly it would have been detected (Mother of child with AML)*

Efforts taken to visit multiple doctors were often delayed due to geographical issues, especially for those residing far away from diagnostic or treating facilities. Travelling long distances with a sick child was uncomfortable for the family, especially with unreliable public transport systems. Few families had their own transport, but difficult terrain often aggravated the situation.

#### Determinants influencing the referral pathway

Suggestions were offered by relatives, friends or strangers to seek various medical advice, influencing caregiver health care-seeking behaviours across all hospitals. Most families sought allopathy although few were first influenced to seek treatment through alternate medicine, or visiting the RMP or traditional healer:

*Yes the local priest in the village. We got the treatment done through him. Then they had made some medicines and they also did a plan to exorcise jaundice (…) Then in the end I spoke to my family members and showed him to an Ayurvedic doctor in the area. (Father of child with ALL)*

Sometimes the opposite advice fast-tracked the trajectory:

*Actually, when we went to Srinagar’s [tertiary hospital] we met a patient over there. He told me that if you stay here you will not get cured. Go straightaway to Delhi as they have so many hospitals over there. The treatment will be done immediately. (Uncle of child with ALL)*

Mistrust or scepticism of the health system throughout the referral pathway also contributed to delays. This was mainly expressed by the male caregivers, who had a more pivotal role in seeking care. It is presumed their direct involvement and exposure to opinions and experiences shaped their pre-conceived notions of the health system. For example, a few fathers had perceptions of public hospitals being too overcrowded and involving many processes, while others ignored advice to conduct tests in a private hospital.

*The issue with government hospitals is that apart from trouble what else do you get there? They make you run here and there. (Father of child with Retinoblastoma)*

*So we had ignored, because basically in India sometimes private doctors suggest tests for milking money and all. Everybody knows about it. (Father of child with ALL)*

Poor knowledge of disease and diagnosis/treatment also acted as a barrier to accessing timely care. Despite visiting multiple health care providers along the referral pathway, some caregivers did not recognise the urgency of the situation to pursue immediate care due to paucity of knowledge. Some families were unaware of cancer and those particularly from rural backgrounds or low SES often questioned the disease aetiology:

*I wanted to ask the doctor that since we lie next to him will we also get the same disease? Because the mosquitoes which are biting him are biting us as well. So we should not get the same illness. So this question was coming up in my mind that how can we prevent this illness? Can we also get this infection? (Adult cousin of child with AML)*

Many families were unaware of certain tests, radiation or chemotherapy and few thought of researching the disease and procedures while others were afraid to enquire further. Knowledge impacted caregiver confidence but also showed their naivety, some undermining the seriousness of a cancer diagnosis. Some families were therefore prepared to start treatment immediately but others did not understand the urgency of timely diagnosis and treatment. For some caregivers, knowledge increased upon consulting the treating doctor but was also influenced by other families:

*So only after we came here, we came to know that this is not a very serious illness. If we are careful about the food and hygiene then my brother will become fine again. So that is why I came to [tertiary hospital] because I had heard that people do get cured over here and go back. (Adult sister of child with PNET)*

#### Patient navigation at the treating hospital

Many families experienced problems in navigating the treating hospital across both cities, especially those who were uneducated. This problem was more prevalent in public hospitals as compared to private hospitals. Time intensive bureaucratic tasks were stressful and discouraging for caregivers rushing to commence treatment**.** Time was wasted in locating rooms, long queues, completing administrative procedures such as filling forms, applying for reimbursements and registering for diagnostic tests. One teenage boy suffering from PNET took on the labourious administrative tasks himself, including waiting in long queues as described by his mother:

*We have to wait for 4 to 5 hours at times. My son wakes up at 3am and gets the [registration] number. Then he tells me that mother you eat and come, I will go ahead. (Mother of child with PNET)*

Another mother described her experience in a public hospital before getting her daughter’s bone marrow biopsy done in a private hospital:

*Ma’am if we would take our child over there again, then they would have killed her. Because they give such dates that the person will die before that. (Mother of child with ALL)*

From the narratives, time estimation between symptom onset and starting the treatment ranged from three weeks to a year, with most families reporting a duration of more than 6 months. Few families had limited visits and were diagnosed at tertiary hospitals near their native home place. Some families from middle-upper SES had more connections with specialists and better knowledge of the referral pathways and treating hospitals than lower SES families, and this advanced their progression toward diagnosis and treatment. Referral pathways for children being treated in Delhi hospitals were more diverse than those treated in Hyderabad. Most families from districts in and around Andhra Pradesh and Telangana had Hyderabad tertiary hospitals as the second or third visit, where they often got a definitive diagnosis and treatment. Families from north Indian states however, had more tertiary hospitals to choose from, either in a neighbouring state or close metropolitan city making their journey longer, although most did not have appropriate diagnosis and treating facilities for children with cancer.

### Importance of social supportive care

Although medical care was received intermittently throughout the referral pathway and mainly upon reaching the participating treating hospital, families across both cities often spoke about the need for social support while accessing medical care. Families relied on religious and cultural beliefs (2.1) and it was important to understand the family and social dynamics (2.2) in supporting the primary caregiver financially, emotionally and its subsequent effect on the child. Secondly, upon starting treatment, the ability to stay while receiving treatment (2.3) and health care provider-patient support (2.4) also affected access to medical care.

#### Religious and cultural beliefs

Religious and sociocultural beliefs influenced the type of support families sought throughout the journey. Alternate treatment was sometimes sought upon symptom onset, and re-visited in conjunction to allopathic care. Although this was more prevalent amongst families from rural or low SES, middle-upper class families also sought alternative care such as one businessman who was treating his son in a private hospital:

*We took him to Haridwar to a Vaid* 1*and his medicines are being given to him. Then we are also giving him the paneer* 2*made from the milk of the best cow called Gir Gai. And we are also feeding him the pure urine of that cow’s calf as well. (Father of child with Medulloblastoma)*

All families expressed a degree of relief using religious means and attributed cause of disease to either an ‘evil eye’, fate or God. Religious influence was also predominant in affecting financial support, showing the extent to which religion played a big role in accessing care:

*Among Muslims we don’t even believe in getting insurance done. It is supposed to be illegal. All of that is unnecessary. (Father of child with ALL)*

#### Family and social dynamics

The impact of social and relational roles was evident in the type of support received throughout the journey in both cities. Parents often had to manage other responsibilities apart from taking time out to care for the sick child. Firstly, the opinions of male caregivers regarding the health system (section 1.2) demonstrated that husbands were usually the ones to take the child for health care visits, make decisions and navigate the health system while the female caregivers engaged more in domestic house work. While the shared responsibility might have accelerated the journey, the process was still hindered over time with increasing duties and concerns over the treatment.

Secondly, some caregivers were unable to pursue care due to resource constraints, which were often deliberately withheld by relatives due to domestic conflicts and different set of ideologies. Due to lack of money, one caregiver along with her husband felt it was easier to let her child die naturally believing they could produce more children later who would be healthy:

*They thought of not getting the treatment done since it was their first child. They thought of ignoring it is what I felt. They would have ignored and taken her back and left her to her fate. (Uncle of child with Wilms tumour)*

Upon reaching the treating hospital, caregivers expressed momentary relief regarding the diagnosis and starting treatment and their confidence grew upon seeing other children also receiving similar treatment. However, some were also apprehensive about the hospital environment. With little to no social support, caregivers from other cities expressed confusion, worry and felt uneasy staying in a new city with unknown people, especially those who were uneducated and from rural backgrounds. Some caregivers spoke of situations of receiving no social support from relatives or friends and leaving behind dependent family members:

*The biggest problem is that I have two children whom I have left and come here. My brother is no more. She is my brother’s daughter. I have left everyone and come here since 15 days now. (Uncle of child with Retinoblastoma)*

Others expressed gratitude and the significance of a helping family member or non-medical staff of the hospital.

#### Ability to stay while receiving treatment

A crucial barrier to being able to stay for diagnosis and treatment upon reaching the cancer hospital is finding accommodation. Some caregivers from other cities, particularly those caregivers from a lower SES, were forced to sleep on footpaths, next to metro stations or in government run homeless shelters. This was similar across both cities. The ongoing tests and appointments meant they had to find accommodation close by, and often the child’s health would deteriorate to the point of requiring emergency care. Due to this, even though some families were offered accommodation on the outskirts of the city, they refused it.

*I spend the day here and I stay on the road at night (…) I am lying here till evening and I don’t even get food to eat. I don’t get a chance to bathe for 2-3 days at a stretch. (Father of child with Rhabdomyosarcoma)*

Once treatment began, caregivers are recommended to provide home cooked food to the patient, which proved challenging for those without appropriate accommodation or facilities. Families including the sick child then resort to eating local unhygienic street food, worsening the condition:

*We couldn’t even change our clothes here properly. We would wear the same two sets of clothes regularly for 5 days (…) Today in the morning we had rice and the rice was such that when we made balls out of it and threw it then it started bouncing like plastic. So I don’t know if the rice was made of plastic or not. So that is why we are managing with whatever we are getting. We had thought that if we get a place to stay then we [could store] flour and water and everything. (Adult cousin of child with AML)*

#### Health care provider-patient support

Some caregivers recounted negative experiences from certain health care providers during the referral pathway, where the doctor’s dismissive attitude and inconsistent referrals made them feel insignificant and helpless to progress through the journey:

*They gave us too much trouble. She was in a lot of pain. When I would take her to emergency they would say why have you got her here. Take her there! (…) They said they cannot do anything more for her so take her away. (…) [Then] we showed in [out-patient department] but they said go back and show her in emergency. This is how they behaved with us. (Mother of child with ALL)*

Upon reaching the treating hospital, the doctor-caregiver relationship was again a recurring matter, where some caregivers expressed confusion, lack of trust and disappointment in health care providers in selected hospitals, while others were satisfied. Upon paying for treatment, a few caregivers appreciated the government or private health insurance schemes available to them, while others were dissatisfied with the application process or financial coverage received. Non-governmental organisations (NGOs) and social worker teams in all hospitals played an important role in supporting those families who were aware of these services. However, unfortunately not all caregivers were aware of the existence of these NGOs.

### Accumulative impacts of the journey

There was a clear connection between pathways taken and its accumulated impacts upon families in both cities. Financial impact (3.1) was a common problem for all families which affected the time taken, social relations and quality of care. Ongoing emotional and psychological impacts of the pathway (3.2) refers to the experiences of the caregiver and child resulting in a variety of emotions which facilitated or depleted the will to persevere.

#### Financial impacts

Financial problems were common for families across both cities. Few families from a middle-upper SES had full or part insurance for their child. Most families who live below the poverty line obtained free or subsidized treatment from state or national government schemes, however some families first had to pay upfront. To pay all expenses most caregivers reported selling off livestock or personal properties and borrowing money or taking a loan. In addition, all caregivers who were the primary breadwinners of the family, had to take leave from work resulting in a loss of income:

*He sells vegetables on the cart. The work mainly happens in the evening and the radiation also takes place mainly in the evening. So that becomes a problem because we have to leave the work and come here (Adult cousin of child with Brainstem Glioma)*

Caregivers who were dependent on their spouse’s income faced an additional challenge of leaving behind other children to bring the sick child for treatment. Financial impacts were also felt due to external influences, such as the Indian government’s 2017 demonetization policy, where certain Rupee notes became invalid to use, resulting in families unable to spend money to access care. Many caregivers from middle-upper SES acknowledged and empathised with families of lower SES who had financial problems.

#### Ongoing emotional and psychological impacts of the pathway

Some families recalled sensitive incidents during the referral pathway that burdened them psychologically, especially due to the relationship dynamics between health care providers and families. One father described the situation of being referred back and forth between two hospitals with his daughter who was suffering from extreme body pain:

*After going there I felt my condition was like a dog. The child was in so much difficulty and was just wrapped in a towel the entire night crying. (Father of child with ALL)*

Impacts were also felt beyond the patient’s family, where additional support was sometimes offered from the community. However, negative psychological and emotional impacts were common across all caregivers interviewed in both cities. Worry, especially over the disease diagnosis, was expressed by all caregivers who understood the diagnosis of cancer:

*So I showed the doctor the report and they told me this was the case. I got really scared and started feeling dizzy and I fell. (Mother of child with Non-Hodgkin Lymphoma)*

In addition, negative influences from either family or community also placed an added burden upon caregivers:

*Others say that this cannot be cured. Why do they say so? They should give the confidence that it can be cured. What has the child done? It’s not fair for such small innocent children to bear this pain. Sometimes I feel instead of seeing this, the child should not have arrived into the world. (Mother of child with Ewing’s Sarcoma)*

Caregivers also expressed concern for their own health in many interviews as well as the emotional and psychological impacts upon the child, where older patients who could comprehend the situation expressed their anguish more apparently:

*I said Sir please check her (…) I am not lying. She is in a lot of pain. So he said that I cannot admit her and just referred her to a neurologist saying that the child has some problem with her brain (…) The child started crying, she started saying that mother even though I am not crazy they will definitely make me mad now. So I said forget it child, don’t worry. We will consult someone else. (Mother of child with ALL)*

## Discussion

The journey to accessing appropriate health care for children with cancer in India is multifaceted and there is a labyrinth of pathways. Reflecting the realities of participants’ lives, the themes delineated within the data are interconnected even though each experience was unique. Families in north India travelled greater distances to Delhi than those traveling to Hyderabad. The duration of interviews was also longer for participants in Delhi. These participants narrated more experiences coming to Delhi which consisted of more complex pathways and health care provider visits compared to participants in south India. Cultural diversity of expression between north and south Indians may also be a contributor to varied narratives. Families in public hospitals were mostly from a lower SES and experienced more barriers compared to families receiving care in private hospitals or of higher SES. Although we did not seek to capture SES data using any standardised instruments, the caregiver occupation, narratives of situations where families had literacy difficulty and financial barriers were evidence enough for distinguishing families from lower and middle/upper socioeconomic backgrounds.

Time intervals along the path to appropriate care were influenced by the number of visits to various health care providers, the type of services available and the quality of social support offered, which in turn was influenced by the cumulative financial and emotional impacts. The occurrence of misdiagnoses during the symptom onset stage may be reflective of poor awareness and sensitization of childhood cancers by general practitioners leading to a delay in diagnosis [16]. The lack of essential diagnostic facilities at certain provider visits contributed to erratic referral pathways, highlighting the weak health system structure in regional areas, forcing families to seek care further away from home. This inadequacy in service availability coupled with a mistrust in the health system, contributed to greater delays. Perceptions that some hospitals fare better than others, or that hospitals would have fastened the diagnostic pace compared to smaller private clinics, were examples of mistrust and scepticism of the system which could have also potentially hindered the decision making process of care-seeking. In addition, even upon reaching the treatment centre, patient navigation issues within the hospital due to administrative and bureaucractic tasks or long waiting times, showed that health system barriers continued to impede the journey to timely care.

The complexity of the system and emotional burden of the process means that pursuing care pathways required considerable resilience, clarity of thought, patience and motivation by caregivers. Hence, an important part of strengthening the health system for childhood cancer care (and a pivotal theme in our findings) is the need for psychosocial and supportive care in reducing the undesirable effects of delay and the negative impacts of the diagnosis on emotions and resources. This has also been reflected in other settings highlighting the need to reduce a universally distressing and potentially traumatic experience for all families [23–26].

The demands of the referral pathway depleted caregiver capacity to engage positively, impacting on early commitment to treatment success and impeding their overall access to appropriate care. The amount of emotional, social, financial and informational support required was correlated with time taken to reach definitive diagnosis and care. A systematic review by Brocken et al. (2012) revealed that rapid diagnostic pathways reduced the period of diagnosis-related distress and showed an “absence of a detrimental effect on anxiety” [27]. Although the study focused on adult patients, it showed reduced period of uncertainty and better patient satisfaction through “one or two-stop diagnostic services” [27]. Such interventions can be equally useful for diagnosed children, positively impacting time taken.

Apart from suggestions provided by friends or relatives to quicken the journey, caregivers did not mention any additional information of benefit provided by health care providers during the referral pathway. Moreover, referrals were sometimes done in a manner which was dismissive or obscure, leading to pre-conceived notions of the health system (as mentioned above) and caregiver demotivation. Blazin et al. (2018) reported that effective communication skills by health care providers fostered comfort and better coping mechanisms for families throughout the journey [28]. Mack et al. (2007) also reinforced that even if health outcomes were poor, parents in the US experienced hope when prognostic information was appropriately communicated [29]. A positive experience with health professional attitudes prior to treatment was largely not the case in our results and this varied upon treatment experience between public and private hospitals. However, apart from behavioural interactions, family dynamics in the Indian culture may also lead to improper patient-provider relations. An example is the phenomenon of ‘collusion’, where upon one caregiver’s request, health care providers may withhold diagnosis information to other caregivers involved, leading to varied patient-provider experiences [30].

Understanding the context of the caregiver’s social and relational circumstances during the journey was critical. A general common barrier amongst caregivers was the poor knowledge and awareness of the disease and treatment, reflecting the need for education efforts and community sensitisation towards childhood cancers. However, if caregivers understood the seriousness of the diagnosis earlier on and had the resources, it is likely that social/relational responsibilities would still impede caregiver ability to singularly focus on the medical journey. It was also important to note that men mostly navigated the health system to seek care, while women were culturally expected to raise the children and stay at home attending to the child and domestic tasks. While this was not the case for all families, the cultural predisposition highlighted a deficit on successful care-seeking pathways.

Many families expressed momentary relief of obtaining a final diagnosis and commencing treatment upon reaching the treating hospital, receiving help from NGOs and social workers and upon seeing other families facing the same battle. This was reflective of the exhaustion and fear experienced during the referral pathway and an indicator of the solitary nature of this journey. Others expressed frustration which reflected the cumulative effects experienced, especially when recalling sensitive incidents during the referral pathway, influencing their engagement during the treatment phase. During interviews, many caregivers cried while recalling what had occurred yet feeling inclined to focus on the present needs given the timing of the interview. This illustrated the emotional intensity in shaping present experiences. It again draws the dire need of enhancing quality of care through compassionate social workers, doctors and NGOs. These small acts of compassion were indeed recognised in a few caregiver narratives and must not be underestimated for improving ongoing engagement in care.

Referral pathways are intrinsically linked to the treatment experience and is also important in addressing abandonment of care which is relatively common in LMICs [31]. When the topic of access to care is examined, it is often examined with referrals prior to diagnosis and treatment as separate. Psychosocial supportive care deficits are often unseen or viewed separately to the treatment experience but are an important part of the entire care continuum. While referral pathways cannot be improved by specialist cancer treatment hospitals alone, there can be greater recognition of the family’s emotional and resource depletion that occurred along the journey to diagnosis, which may positively affect the way health services are provided and received upon treatment commencement. It is worth pointing out that this study was designed to focus on barriers to accessing care. We believe that despite these barriers, there are strengths in the system as well. This is because the system continues to assist and manage a large number of patients in many centres, with minimal cost to patients due to government support.

## Conclusion

This qualitative study provides an in-depth understanding and evidence to further explain the problem identified through quantitative research on time intervals for childhood cancer treatment in India. It highlights the impacts of the referral pathways upon the patient’s and family’s treatment experience. Limitations of this study were that we sought to understand experiences from symptom onset up to the initial stage of treatment and were unable to capture the experiences of families undergoing treatment throughout the cancer journey. Future research could expand to follow families into their treatment. In addition, it is also important to recruit and study experiences of those children and families who never reached the treatment centre. However, we believe that most of the barriers identified in this study would also apply to those patients who never started treatment. We acknowledge that despite our best efforts to ensure participant confidence in narrating stories within the hospital premises, there is a possibility with such studies that respondents may not share all the information or may modify it. It is therefore imperative that studies ensure fidelity of the research by investing time in the participant information process to gain participant confidence before letting them narrate the experiences. The need for support was not only evident through narratives, but also through caregivers who expressed gratitude post interviews for giving them an opportunity to narrate their experiences. This study highlights the need for hospital based counselling services for families. While collective support can either enable or disable a referral pathway, improving access for childhood cancers should be defined as both timely and less traumatic.

## Supplementary information

**Additional file 1: Table S1**. Barrier themes, sub-themes and codes used in initial iterative analysis. **Table S2.** Caregiver demographics by cancer type, caregiver characteristics and distance travelled to treating city.**Table S3.** Names of Institutional Review Boards which approved the study.

## Data Availability

Full datasets analysed in this study are not publicly available due to risk of individual privacy being compromised.

## Abbreviations

ALL: Acute Lymphoblastic Leukaemia
AML: Acute Myeloid Leukaemia
BPL: Below poverty line
GP: General practitioner
HIC: High-income country
LMIC: Low and middle-income country
NGO: Non-governmental organisation
NHL: Non-Hodgkin Lymphoma
PNET: Primitive Neuroectodermal Tumour
RMP: Rural medical practitioner
SES: Socioeconomic status

## Footnotes

1 practitioner of Ayurvedic medicine

2 cottage cheese
