# Supplementary material for: A health care labyrinth: perspectives of caregivers on the journey to accessing timely cancer diagnosis and treatment for children in India
Source: BMC Public Health. 2019 Dec 2;19:1613. doi: 10.1186/s12889-019-7911-x (PMC6889559; doi:10.1186/s12889-019-7911-x)
Supplement: Supplementary file 1 — Additional file 1: Table S1. Barrier themes, sub-themes and codes used in initial iterative analysis. Table S2. Caregiver demographics by cancer type, caregiver characteristics and distance travelled to treating city.Table S3. Names of Institutional Review Boards which approved the study. [file 12889_2019_7911_MOESM1_ESM.docx]

# **Additional file**

Table S1**:** Barrier themes, sub-themes and codes used in initial iterative analysis

| **Barrier themes and sub-themes** | **Codes** | **Sub-codes** |
| --- | --- | --- |
| Financial constraints   - Lack of personal money to start treatment - Lack of personal money to continue treatment - Lack of personal money for indirect costs such as food, travel, accommodation - Unable to obtain funding and insurance schemes - Funding/insurance schemes provided not adequate - Took a loan - Sold property - Loss of job, daily wages | FIN | FIN_I  FIN_HS |
| Social and cultural beliefs   - Lack of family support - Lack of community support - Fear of engaging with urban communities - Absence of caregiver - Gender bias - Preference for alternative treatment - Duration of treatment | SOC | SOC_I |
| Infrastructure   - Lack of beds for patients - Lack of basic hospital equipment for treatment - Lack of available drugs - Lack of appropriate diagnostic testing facilities - Long waiting times - Little or no psychological support for patients/caregivers - Difficulty in navigating health care system | INF | INF_HS |
| Health facility organization   - Medical staff organization - Methods of peer review - Methods of reimbursement - Hours of operation - Administrative delay - Cancer registry system | ORG | ORG_HS |
| Geographical issues   - Rural areas - Poor roads - Transportation difficulties - Difficult terrain - Distance - Lack of accommodation close to facility | GEO | GEO_I  GEO_HS |
| Service provision   - Lack of trained physicians - Lack of adequate physicians/nurses/social workers - Lack of treatment protocols/wrong treatment protocols - Protocols not followed - High patient load to service delivery facilities - Support programs for patients/caregivers | SER | SER_HS  SER_D |
| Awareness   - Lack of education - Lack of acceptance of condition - Lack of understanding of disease - Caregiver’s attitudes - Health care provider’s attitudes | AWA | AWA_I  AWA_HS  AWA_D |

Note: I = individual barriers, HS = health system barriers, D = disease barriers, FIN = financial, SOC = social, INF = infrastructure, ORG = health facility organisation, GEO = geographical, SER = service provision, AWA = awareness.

Table S2: Caregiver demographics by cancer type, caregiver characteristics and distance travelled to treating city.

| **ICCC** | **Cancer type of patient** | **Caregiver type** | **Status** | **Distance for families living outside Delhi** | **Distance for families living outside Hyderabad** |
| --- | --- | --- | --- | --- | --- |
| ICCC1 | ALL, AML | Mother, Father, Uncle, Cousin | BPL, Low SES but not BPL, M/U | 1600km (n=8) | 400km (n=3) |
| ICCC2 | Non-Hodgkin Lymphoma | Mother | BPL | - | - |
| ICCC3 | Brain stem glioma, Medulloblastoma | Uncle, Father | Low SES but not BPL, M/U | 30-40km (n=2) | - |
| ICCC4 | Neuroblastoma | Mother, Father | BPL, M/U | - | 100-200km (n=3) |
| ICCC5 | Retinoblastoma | Mother, Father, Uncle | BPL, M/U | 1000km (n=2) | - |
| ICCC6 | Wilms tumour | Uncle, Father | Low SES but not BPL | 1800km (n=1) | 100km (n=1) |
| ICCC9 | Rhabdomyosarcoma | Father | BPL | 900km (n=1) | - |
| ICCC8 | PNET | Mother & Sister | BPL | 1100km (n=1) | - |

Note abbreviations: Acute Lymphoblastic Leukaemia (ALL), Acute Myeloid Leukaemia (AML), Primitive Neuroectodermal Tumour (PNET), Below Poverty Line (BPL), Socioeconomic status (SES), Middle SES (M), Upper SES (U).

Table S3: Names of Institutional Review Boards which approved the study.

1. Institutional Ethics Committee of Max Super Speciality Hospital
2. Institutional Ethics Committee of Indraprastha Apollo Hospital
3. Institutional Ethics Committee of Institute Rotary Cancer Hospital, All India Institute of Medical Sciences
4. Institutional Ethics Committee of Basavatarakam Indo American Cancer Hospital
5. Institutional Ethics Committee of MNJ Cancer Hospital
6. Institutional Ethics Committee of Rainbow Children’s Hospital
7. Institutional Ethics Committee of All India Institute of Medical Sciences
